# Temporal transcriptomic profiling of the ant-feeding assassin bug *Acanthaspis cincticrus* reveals a biased expression of genes associated with predation in nymphs

**DOI:** 10.1038/s41598-017-12978-0

**Published:** 2017-10-04

**Authors:** Fei Kou, Hu Li, Shujuan Li, Huaizhu Xun, Yinqiao Zhang, Ziqiang Sun, Xuguo Zhou, Wanzhi Cai

**Affiliations:** 1 Key Laboratory of Pest Monitoring and Green Management, Ministry of Agriculture, Department of Entomology, China Agricultural University, Beijing, 100193 China; 2 Maricopa Agricultural Center, University of Arizona, Maricopa, AZ 85138 USA; 3 Department of Entomology, University of Kentucky, Lexington, KY 40546-0091 USA

## Abstract

*Acanthaspis cincticrus* (Stål) is an assassin bug with a specialized camouflaging behavior to ambush ants in the nymphal stages. In this study, we comprehensively sequenced all the life stages of *A. cincticrus*, including the eggs, five nymph instars, female and male adults using Illumina HiSeq technology. We obtained 176 million clean sequence reads. The assembled 84,055 unigenes were annotated and classified functionally based on protein databases. Among the unigenes, 29.03% were annotated by one or more databases, suggesting their well-conserved functions. Comparison of the gene expression profiles in the egg, nymph and adult stages revealed certain bias. Functional enrichment analysis of significantly differentially expressed genes (SDEGs) showed positive correlation with specific physiological processes within each stage, including venom, aggression, olfactory recognition as well as growth and development. Relative expression of ten SDEGs involved in predation process was validated using quantitative real-time PCR (qRT-PCR).

## Introduction

**Figure 1:**
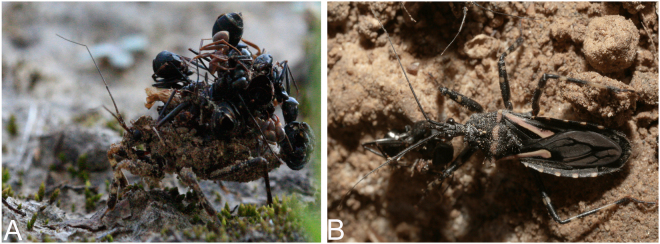
The nymph and adult of *A. cincticrus*. (**A**) A masked nymph camouflaged with ant corpses, dust and soil particles. (**B**) An adult male. Photographs were taken by F.K.

*Acanthaspis cincticrus* (Stål) (Hemiptera: Reduviidae) is a predatory assassin bug, which feeds on ants and can be found in the vicinity of ant nests, waiting for the preys^1^. This species is native to Oriental Region with one generation per year. Nymphs of this species have five instars and exhibit both natural and corpse camouflaging behaviors^1^. They cover themselves with a range of materials found in their environment, including ants corpses and other insects, dust and soil particles, which they affix on themselves with the viscid secretions from specialized setae on the abdomen^2^. Camouflaging in *A. cincticrus* is specific to the nymphs (Fig. 1A) and is absent in the adults (Fig. 1B). This behavior has been documented in some neuropterans^3^. Camouflaging may benefit nymphs in two ways: by being less visible in stalking their preys and by not being obvious to potential predators. While studies on *A. cincticrus* thus far have focused on the morphology, biology and behaviors related to camouflaging in nymphs^1,2^, the molecular mechanisms underlying development and predation are unknown.

In predatory insects, arousing, paralyzing and sucking are important for predatory process^4^. These processes are influenced or regulated by several genes or pathways involved in the identification of chemical signals, and in the regulation of neuromodulators for agonistics and the digestion of the prey^5–7^. Predators locate and interact with their prey by using species-specific semiochemicals, such as the detection and discrimination of odorant-binding proteins (OBPs) and chemosensory proteins (CSPs)^5^. After locating the target, aggression is displayed by predaceous arthropods against their prey^8^. Factors that affect aggressiveness and/or fighting success in predaceous arthropods include body size^9^ and contestants’ age^10^. Previous studies reported that many genes, such as *cyp6Q20*, *tyramine receptor*, *octopamine receptor* and *metabotropic glutamate receptor B*, were likely to be involved in the regulation of complex behavioral phenotypes^11,12^. *Tyramine receptor* and *octopamine receptor* serve as aggression modulating neurotransmitters that affect excessive aggressiveness and impulsiveness in the biogenic amine signaling pathway^11^; *metabotropic glutamate receptor B* is a part of G protein coupled metabotropic receptors family, which is the major excitatory neurotransmitter acting through multiple independent receptors, involved in regulating aggressive behavior of mice^12^.

The paralyzing and digestion process of assassin bugs includes extra oral digestion (EOD)^13^ which is a chemical pretreatment to mobilize nutrients and is common in the predation of Heteroptera^7^. Venom and/or digestive enzymes have a biochemical role of typically stunning, killing prey and even aiding in prey digestion^7,14^. The venomous saliva of predatory reduviid bugs is known to contain a complex mixture of proteins^15^, peptides^16^ and enzymes^17^. The most abundant enzymes that are present in the salivary secretion include proteinases, phospholipase, trypsin like enzymes, esterase, serine proteases, etc^18^. Serine proteases, found in many organisms, have attracted broad interests because they have diverse physiological functions that affect processes such as digestion, immune response, cellular differentiation and prothrombin activator^7,13^. The presence of trypsin-like enzyme in the salivary glands of *Zelus renardii* indicates that it has evolved or retained *trypsin-like* genes for protein digestion^7^. Although most reduviids are common predators, studies on the factors influencing the feeding process focused on digestion^7,15–18^.

The transcriptomic and bioinformatic approaches presented in previous studies have been served as a foundation to explore the relationships between gene regulation and behavioral evolution in other species^5,11^. In this study, we performed a comprehensive transcriptome analysis during eight life stages of *A. cincticrus*, including egg, five instar nymphs, female and male adults. We identified 13,479 significantly differentially expressed genes (SDEGs) and also identified and characterized 115 SDEGs involved in predation, indicating differences between genders and among life stages. We performed quantitative real-time PCR (qRT-PCR) analysis to determine the expression profiles of ten SDEGs involved in predation from different life stages. The results could help elucidate the role of venom-related, aggression-related and olfactory-related genes involved in the predation of reduviids.

## Results

### Whole-transcriptome sequencing and annotation of the predicted proteins

**Figure 2:**
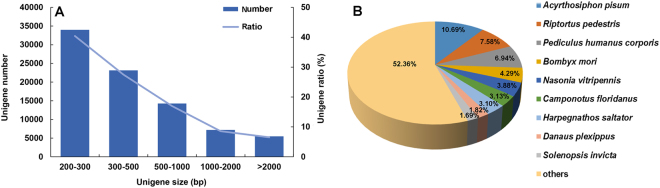
The statistics of assembly and homology analyses. **(A**) Size distribution of unigenes. (**B**) Species distribution of the BLASTX against NCBI-NR database, proportions of more than 1% were shown.

cDNA libraries from all life stages of *A. cincticrus* yielded about 176 million clean sequence reads with an average quality value ≥ 30. GC content of the sample was averaged at 38.03% (Table S1). After assembling the clean reads, we obtained 164,745 transcripts and 84,055 unigenes with an average sequence length of 1,366 bp and 667 bp, and N50 with average lengths of 2,731 bp and 1,087 bp, respectively (Table 1). The length of unigenes ranged from 210 bp to 29,391 bp (Fig. 2A). Of which, 12, 679 (or 15.09%) unigenes had sequence length more than 1,000 bp. The result is similar to the number of unigenes reported in the moths *Dendrolimus punctatus* (70,664)^19^ and *Athetis lepigone* (81,356)^20^. A proportion of unigenes was annotated based on the available protein database for *A. cincticrus*. 24,402 unigenes (or 29.03%) were annotated as coding hypothetical proteins. Homology analysis of *A. cincticrus* unigenes showed that they best matched with the species from Hemiptera, the pea aphid *Acyrthosiphon pisum* and the bean bug *Riptortus pedestris* (Fig. 2B).

**Table 1:** Summary of *Acanthaspis cincticrus* transcriptomes.

Length range	Transcripts	Unigenes
200–300	39,146(23.76%)	33,985(40.43%)
300–500	31,343(19.03%)	23,125(27.51%)
500–1000	29,635(17.99%)	14,266(16.97%)
1000–2000	28,485(17.29%)	7,202(8.57%)
2000+	36,136(21.93%)	5,477(6.52%)
Total number	164,745	84,055
Total length	225,069,311	56,032,206
N50 length	2,731	1,087
Mean length	1,366	667

### Functional classification and Kyoto Encyclopedia of Genes and Genomes (KEGG) analysis

The functions of unigenes were predicted by Gene Ontology (GO) analysis at the macro level. In total, 11,682 unigenes were classified into 58 sub-categories belonging to three GO functional categories: cellular component (CC), molecular function (MF) and biological process (BP) (Fig. S1). The GO classification of unigenes indicated that ‘cell part’ (20.79%) and ‘cell’ (20.68%) were the dominant sub-categories in the CC category, ‘catalytic activity’ (42.40%) and ‘binding’ (38.83%) were the most dominant sub-categories in the MF category, and ‘metabolic process’ (24.23%) and ‘cellular process’ (21.81%) were the most dominant sub-categories in the BP category.

We also annotated the unigenes by searching the Cluster of Orthologous Groups (COG) database to classify the functions of the predicted proteins. A total of 6,822 unigenes had a COG classification. Among the 25 COG categories (Fig. S2), the cluster for ‘general function prediction only’ was the most dominant group (18.25%), followed by ‘translation, ribosomal structure and biogenesis’ (8.66%) and ‘post-translational modification, protein turnover, chaperones’ (8.58%).

In order to identify the biological pathways represented in the transcriptome of *A. cincticrus*, 7,193 unigene sequences were mapped to 193 KEGG pathways (Table S2). The most prominent metabolic pathways were protein processing in endoplasmic reticulum (4.25%), oxidative phosphorylation (3.99%) and RNA transport (3.70%).

### The SDEGs in different life stages

We evaluated the gene expression levels to measure the genetic landscape in different life stages of *A. cincticrus*. We performed nine comparisons of the transcriptomes from pairs of life stages (e.g. egg vs 1^st^ instar, 1^st^ vs 2^nd^ instar, 2^nd^ vs 3^rd^ instar, 3^rd^ vs 4^th^ instar, 4^th^ vs 5^th^ instar, 1^st^ vs 5^th^ instar, 5^th^ instar vs adult male, 5^th^ instar vs adult female, male vs female adults) (Table S3). The results yielded 13,479 SDEGs (Table S4), which were then hierarchically clustered in a heat map (Fig. S4). In this heat map, the gene expression profiles differed through all life stages with each comprising a main cluster of high expression genes. In the egg stage, many crucial embryogenesis related genes also comprised maternal genes such as *kruppel*, *hunchback*, *nanos-like protein* and *serine protease snake-like*, *protein takeout-like*, *maelstrom*, *pumilio homolog 1-like* and *staufen* (Table S5). The nymph and adult stages also had their own high expression unigenes, including *cuticular proteins*, *histone H2A*, *cytochrome P450s*, *ecdysone-induced protein* and *juvenile hormone binding proteins* in nymph stages, while *vitellogenin* and *sperm flagellar protein 1-like* in the adults. A previous study reported that these maternal genes, e.g. *snake* and *nanos*, might be involved in the embryonic development of *Oncopeltus fasciatus* ^21^.

The distribution of upregulated, downregulated and uniqueness of SDEGs in the different comparisons were shown in the Table S6. We also analyzed the changes in gene expression between the nymph stages and displayed the distribution of SDEGs using a Venn diagram that illustrated the intersection between the expressed genes in various developmental nymph stages (Fig. S4). In total, 38 SDEGs were shared by all nymph stages. 203, 937, 333 and 614 SDEGs were uniquely expressed in 1^st^ vs 2^nd^ instar, 2^nd^ vs 3^rd^ instar, 3^rd^ vs 4^th^ instar, and 4^th^ vs 5^th^ instar indicating that different life stages had different highly expressed unigenes that participated in specific life activities.

In the comparison of egg vs 1^st^ instar, upregulated SDEGs with the high variation (10+ fold) were *serine protease*, *trypsin-like protease*, *OBPs*, *cuticular protein*, etc. The downregulated SDEGs (3+ fold) were annotated to *early cuticle protein 6*, *hunchback*, *nanos-like protein*, *serine protease snake-7*, *vitellogenin receptor*, etc.

In the comparisons of 1^st^ vs 2^nd^ instar and 2^nd^ vs 3^rd^ instar, the most enriched GO terms were oxidation-reduction process (BP) and structural constituent of ribosome (MF). Lipid particle (CC) and cytoplasm (CC) were respectively enriched in 1^st^ vs 2^nd^ instar and 2^nd^ vs 3^rd^ instar (Table S7). Oxidative phosphorylation, ribosome and lysosome were the top three enrichment pathways in the KEGG pathway analysis (Table S8). When comparing 3^rd^ vs 4^th^ instars, most upregulated genes were enriched for GO categories including protein metabolic process (BP), intracellular (CC) and catalytic activity (MF) (Table S7). According to KEGG enrichment analysis, the upregulated genes were enriched for pathways such as arginine and proline metabolism, wnt signaling pathway and glutathione metabolism (Table S8). When comparing 4^th^ vs 5^th^ instar, most of the SDEGs were categorized into nucleosome assembly (BP), intracellular (CC) and catalytic activity (MF) (Table S7). The KEGG analysis indicated that arginine and proline metabolism, glutathione metabolism and progesterone-mediated oocyte maturation were the top enriched pathways (Table S8).

We also performed a comparison of 1^st^ vs 5^th^ instar. The upregulated genes with the high expression variation (5+ fold) were *pupal cuticle protein precursor*, *defensin*, *histone*, etc. The downregulated genes with high expression variation were *ecdysone-induced protein*, *juvenile hormone binding protein-like precursor*, etc.

Between the 5^th^ instar nymph vs adult male transcriptomes, the upregulated genes with the high expression variation (5+ fold) included *venom serine protease*, *odorant-binding protein*, *methylmalonyl-CoA decarboxylase*, etc. The downregulated genes included digestive and metabolic genes such as *histone H2A*, *prostaglandin reductase*, *cytochrome P450 monooxygenase CYP6* *X* *1v1*, etc.

When comparing female vs male adults, the upregulated genes (5+ fold) included *venom serine protease*, *vitellogenin receptor*, etc. The downregulated genes included *sperm flagellar protein 1-like*, *ejaculatory bulb-specific protein 3*, *heat shock protein 60*, etc.

**Figure 3:**
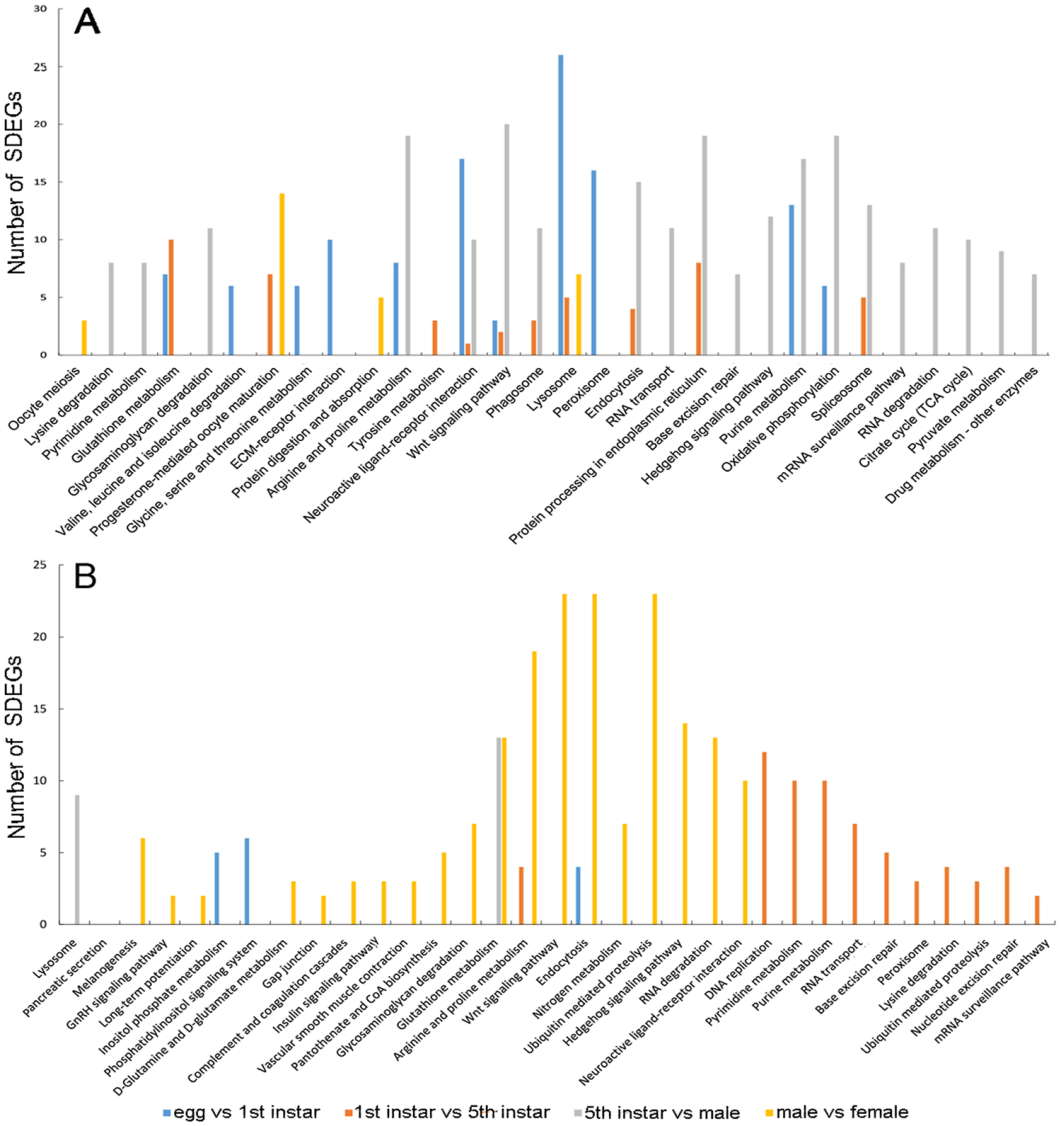
Enrichment KEGG pathways for the upregulated (**A**) and downregulated (**B**) SDEGs in comparisons of different life stages.

The three comparisons (egg vs 1^st^ instar, 1^st^ vs 5^th^ instar, 5^th^ instar vs adult male), including two developmental transitions (eggs to nymphs, nymphs to adults), shared two pathways for the upregulated SDEGs: wnt signaling pathway and neuroactive ligand-receptor interaction (Fig. 3A), which are important pathways in processing information^22^. Three pathways, including arginine and proline metabolism, purine metabolism and oxidative phosphorylation (Fig. 3A), were enriched in two comparisons (egg vs 1^st^ instar, 5^th^ instar vs adult male). The oxidative phosphorylation pathway plays a critical role in the supply of energy^23^. The enrichment analysis indicated that energy metabolism increased during development in *A. cincticrus*. In addition, other genes in this pathway encoding transport oxidoreductase and heat shock proteins (*hsp*s) were upregulated as well. Glutathione metabolism was downregulated in two comparisons (5^th^ instar vs adult male, adult male vs adult female), and arginine and proline metabolism were downregulated in two comparisons (1^st^ vs 5^th^ instar, adult male vs adult female). Inositol phosphate metabolism and phosphatidylinositol signaling system were downregulated in egg vs 1^st^ instar nymph (Fig. 3B). The changes in phosphatase in *Triatoma* saliva could interfere with anti-haemostatic response and facilitate their feeding^24^.

In the GO enrichment of BP, the most enrichment of upregulated SDEGs, including metabolic process, cellular process, single-organism cellular process, signal transduction, proteolysis, and oxidative phosphorylation, were shared by two comparisons (egg vs 1^st^ instar and 5^th^ instar vs adult male) (Fig. S5A). The most enrichment of upregulated SDEGs in the CC (Fig. S5B) included integral component of membrane, cytoplasm, membrane, lipid particle, plasma membrane and microtubule associated complex, were shared in two comparisons (egg vs 1^st^ instar and 5^th^ instar vs adult male). As for the MF (Fig. S5C), two comparisons (egg vs 1^st^ instar and 5^th^ instar vs adult male) revealed five common GO enrichment categories of upregulated SDEGs, including catalytic activity, binding, transferase activity, nucleic acid binding and ATP binding. Four comparisons (egg vs 1^st^ instar, 1^st^ vs 5^th^ instar, 5^th^ instar vs adult male, adult male vs adult female) shared one common GO term (catalytic activity). Serine protease, involved in general digestion and protein metabolism^7,25^, was identified in catalytic activity indicating that the organism was undergoing continuous growth.

Together, the KEGG pathway and GO enrichment analyses identified a diverse group of development-related genes that undergo differential transcriptional responses during the developmental stages and predation process. These genes include maternal genes, cuticular proteins, metabolite synthases, hormone-related genes (Table S5), venom-related genes, aggression-related genes and olfactory-related genes (Table S8).

### Genes putatively involved in predation

**Figure 4:**
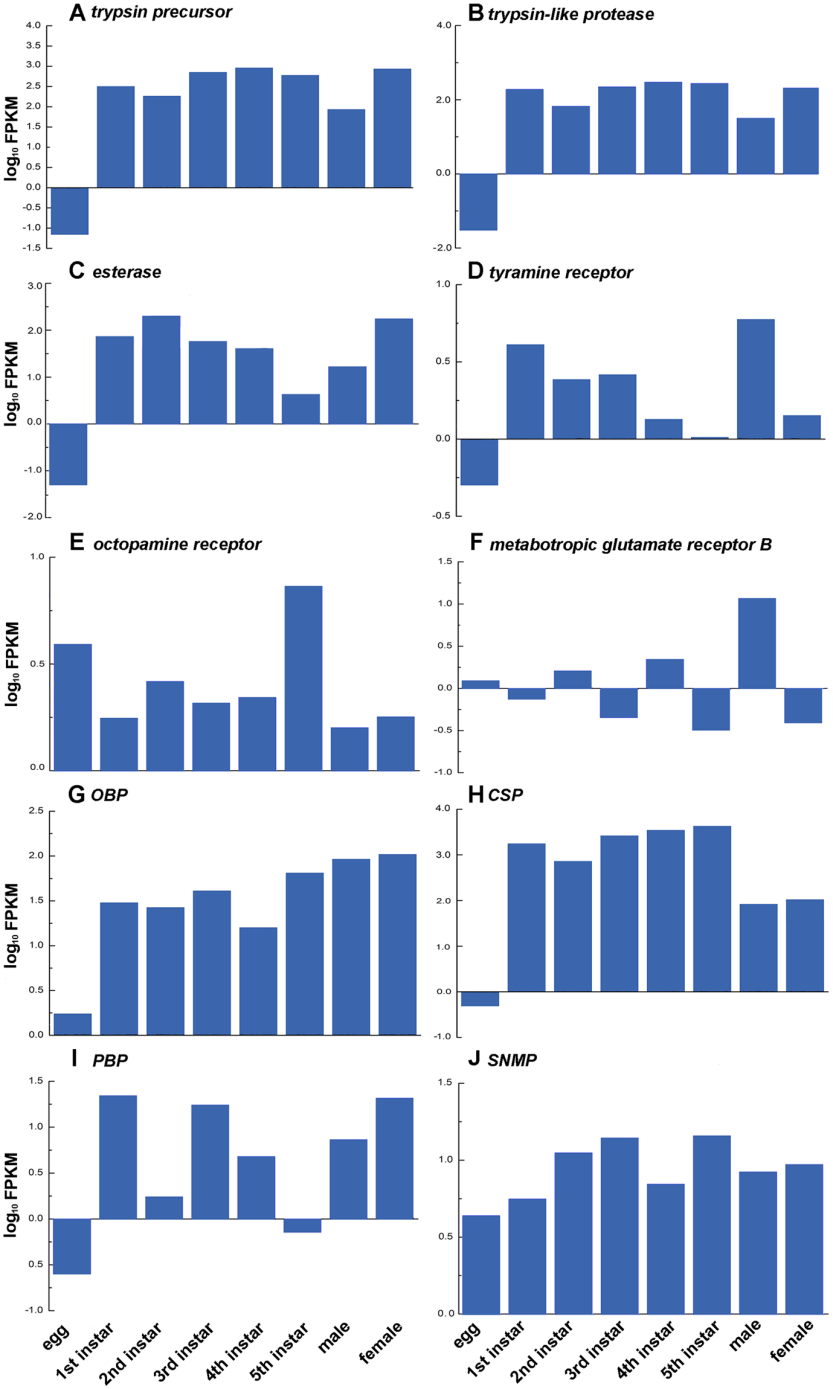
Temporal distribution of ten SDEGs involved in predation. (**A**–**C**) Three venom-related genes. (**D**–**F**) Three aggression-related genes. (**G–J**) Four olfactory-related genes. The transcript levels of SDEGs were calculated as the log_10_ FPKM of each comparison in transcriptome analysis and were shown on the y-axis.

By comparing the transcriptomes from different life stages, we identified 115 genes involved in predation, including 37 venom-related, 23 aggression-related and 55 chemosensory-related genes (Table S9). The venom-related genes included *esterase*, *trypsin-like protease*, *trypsin precursor*, *lipase*, *venom serine protease 34-like*, *serine carboxypeptidase 1*, *lysosomal acid phosphatase precursor*, etc. It appeared that these genes exhibited low expression level in egg stage and relatively high expression level in nymph and adult stages. The transcript levels of *trypsin-like protease*, *trypsin precursor* and *esterase* showed the similar changing trend to that of the most venom-related genes, except *esterase* being low in 5^th^ instar (Fig. 4A–C). The majority of these genes were enriched in hydrolase activity, catalytic activity and peptidase activity.

The aggression-related genes included *tyramine receptor*, *octopamine receptor*, *cyp6Q20*, *deaf1*, *metabotropic glutamate receptor B*, *progesterone*, *androgens*, *serotonin*, etc. Apparently egg had the relatively low expression of these genes compared to other stages. As in the nymph stages, no obvious trends were observed regarding the expression of most aggression-related genes. 5^th^ instar nymph had the relatively low expression of *tyramine receptor* (Fig. 4D) and highest expression level of *octopamine receptor* (Fig. 4E). *Androgens* and *serotonin* with a significant downregulated expression in the 3^rd^ instar. Adult male had the higher expression of most aggression-related genes, e.g. *tyramine receptor*, *metabotropic glutamate receptor B* (Fig. 4F), *deaf1*, *trp*, *progesterone*, *androgens*, and *serotonin* (Table S9), than adult female. The enrichment GO categories were signal transducer activity, cell communication and G-protein coupled receptor signaling pathway.

We identified 22 *OBPs*, 17 *CSPs*, 11 *olfactory receptors* (*ORs*), four *sensory neuron membrane proteins* (*SNMPs*) and one *pheromone-binding protein* (*PBP*)-like transcripts. *OBPs* and *CSPs* exhibited relatively low expression in egg stage and high expression in nymph and adult stages (Fig. 4G,H). *PBP* exhibited relatively low expression in egg, 2^nd^ instar and 5^th^ instar, and showed high expression in the other life stages (Fig. 4I). *SNMPs* seemed to have a constant expression during all life stages (Fig. 4J). Adult male had the higher expression levels of most *ORs* than other stages. These genes were mainly enriched for odorant binding, response to stimulus, phosphorylation as well as in the olfactory receptor pathway.

### Trend analysis

To understand the expression patterns of the 13,479 SDEGs, gene data from all life stages were clustered into 50 model profiles (Fig. S6). 4,183 genes represented 13 significant gene expression patterns, and 33 of 115 SDEGs related to predation could be clustered into five profiles with significance (*p* < 0.05). The genes assigned to most profiles showed variation in nymph stages and demonstrated a bias between genders, e.g. *trypsin precursor*, *trypsin-like protease* and *esterase* showing a bias towards females; *metabotropic glutamate receptor B* and *ORs* was observed as a male-biased expression.

### qRT-PCR validation

**Figure 5:**
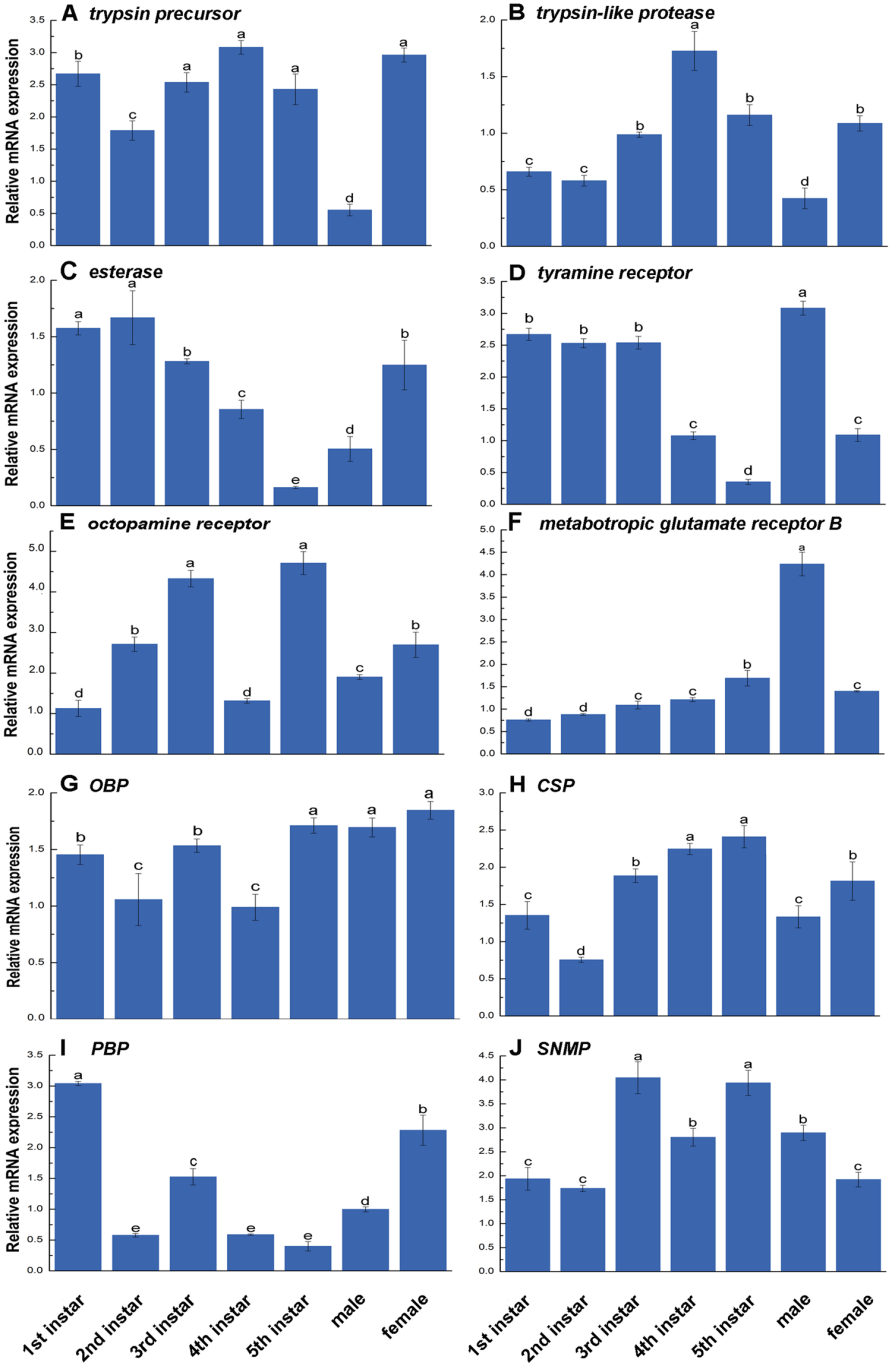
The dynamic expression patterns of ten SDEGs by using qRT-PCR. (**A**–**C**) Three venom-related genes. (**D**–**F**) Three aggression-related genes. (**G**–**J**) Four olfactory-related genes. The relative transcript level of each SDEGs was shown as the mean ± SE. Significant differences among different life stages were indicated by different letters (*p* < 0.05, *t*-test).

To further confirm the quality of the transcriptome, we compared the expression patterns of the seven libraries, including those from 1^st^ to 5^th^ instar nymphs, male and female adults, of ten selected SDEGs (three venom-related, three aggression-related, four olfactory-related genes) involved in predation using qRT-PCR (Fig. 5). The expression trend of seven SDEGs in qRT-PCR analysis was consistent with that detected in the transcriptome analysis earlier (Figs 4 and 5). Inconsistencies were found in the three SDEGs, *tyramine receptor*, *OBP* and *SNMP*, which were different in some stages between qRT-PCR and transcriptome analysis. This difference in gene expression might be caused by the difference in the accuracy of these two assay methods. The SDEGs analyzed in this study were unigenes obtained from transcriptome that were assembled and mapped, while qRT-PCR showed a lower sensitivity than transcriptome sequencing. Nevertheless, qRT-PCR analysis confirmed the direction of change detected by transcriptome analysis, indicating that our results are reliable.

## Discussion

In this study, we sequenced and characterized the transcriptome from the different life stages of *A. cincticrus*, with a particular focus on SDEGs involved in the development and the predation process. The transcripts displayed a variety of interesting differential expression patterns observed across all life stages. The characteristics of gene expression patterns between life stages and predation related unigenes were discussed below.

### Genes involved in development

During its life cycle, *A. cincticrus* undergoes incomplete metamorphosis. Many crucial genes and transcriptional factors, such as *nanos-like protein*, *hunchback* and *serine protease snake-like*, participate in embryonic development of model organisms. For instance, the *nanos* and *snake* gene are core components of the germplasm and are involved in the formation of anterior-posterior body axis during early embryogenesis^21^. These genes were categorized in the GO terms of ‘catalytic activity’ and ‘hydrolase activity’, which are the essential first steps in the eggs activation cascade^26^. In this study, the high expression of genes related to embryonic development revealed the ongoing drastic cellular differentiation process in the eggs.

The nymphs should continually prey on ants to sustain the energy for molting through five nymph stages. They begin movement right after hatching from the eggs and feed on ants to store energy for development and various activities. Genes participating in muscle growth^27^ including *histone H2A* and genes pertaining to metabolic detoxification^28^ including *glycosidases* and *cytochrome P450s* were identified in the transcriptomes of nymphs. *Glycosidases* have been previously proposed to aid in the digestion of endosymbiont cell walls in *Rhodnius prolixus* ^29^. *Cytochrome P450s* are highly expressed in the “oxidoreductase activity”, and are known to be involved in steroid and lipid metabolism^30^ and can participate in insecticide resistance^31^. Insect molting is rigorously choreographed and is coordinated by fluctuations in the juvenile hormone (JH) and ecdysone. JH plays a key role in regulating development and metamorphosis^32^, while ecdysone-regulated genes play key roles during molting and metamorphosis of *Oncopeltus fasciatus* ^21^. We discovered that the expression of *ecdysone-induced protein* and *juvenile hormone binding protein* was high in the early nymphal stages. In general, the wings in reduviids become externally visible during the 2^nd^ instar nymph, and they grow larger at each successive instar, then eventually they are fully developed when reduviids molt into adulthood^33^. We herein found *notch*, which is critical for wing disc development by secreting signal molecules^34,35^, were activated and highly expressed in the 2^nd^ instar nymph of *A. cincticrus*.

In the final stage of development, adults reach sexual maturity followed by the behaviors like courtship, mating and oviposition. The *hsps* in *Drosophila* ^36^ potentially mediate the cellular response to thermal stress and indirectly influence the downregulation of sex steroid receptors, resulting in repressing the proliferation of growing follicles in proestrus, and therefore impact the organism’s fecundity and longevity^37^. Expression of *hsps* was lower in adult female than adult male. The KEGG enrichment term “oocyte meiosis” was enriched among the upregulated genes in the comparison of transcriptomes from male vs female adults, with *vitellogenin* highly expressed in the females. *Vitellogenin* encodes the vitellin in oocytes, and is expressed as an egg yolk precursor in female insects and sterile worker bees, as well as in other insects^37^.

### Genes involved in predation

The predatory process of reduviids involves the following steps: arousing, locating, approaching, paralyzing, sucking, releasing and cleaning^4^. Neuroactive ligand-receptor interaction, wnt signaling pathway, glutathione metabolism, arginine and proline metabolism are important metabolic pathways in managing information and protein digestion. These processes are coordinated by a series of genes. SDEGs involved in predation were enriched in these pathways in nymphs and adults.

Because reduviids can only ingest liquid food, the salivary secretions play a critical role in paralyzing and feeding of their prey. The toxic protein content of venomous saliva in reduviids varies with respect to males and females^38^. Records of adult female reduviids paralyzing their prey more rapidly than adult males reinstate the above fact^39^. The *esterase* was abundant in the saliva of *A. cincticrus* may likely aid in paralysis of prey^18^. In the salivary glands, *trypsin-like mRNA* is dominant which is involved in cleaving the proteins at lysine and arginine residues^40^. The transcript levels of most SDEGs were low in eggs, relatively high in nymph and adult stages and exhibited a bias towards adult female. This result was consistent with the eggs being in a static state, while the nymphs feed on mass of ants for development and the females reserve energy to oviposition. The females have high expression of *trypsin precursor*, *trypsin-like protease* and *esterase*, leading to the stronger toxicity of the saliva than males and obtaining more ants due to their ability in paralyzing the prey more rapidly than the males. The activation and upregulation of these genes are critical for successful paralyzing and digestion of the prey.

Success rate of aggression can be influenced by several factors, such as physical disparities (e.g. size, strength and weapon)^41,42^, as well as physical exertion and experience in previous fights^43,44^. For example, different size of the parasitoid wasps has a different fighting propensity and weak-fighting ones usually stay hidden to avoid the stress of fighting^45^. Males usually are more likely to respond aggressively than females^10^ due to the limited opportunities to mate^46^. Monoamines, neuropeptides, and pheromones have been implicated as important neuromodulators for agonistic displaying in insects^47^. The expression of *cyp6Q20* in olfactory sensory organs has also been reported to regulate hereditary-related and environmental-related aggression in *Drosophila melanogaste*r^48^, suggesting a function in response to aggression-related stimuli. These results suggest that changes in *octopamine receptor* of nymph during an aggressive confrontation may be related to sufficient amounts of *octopamine* that induce the initiation, level and duration of aggressive behaviors^49^. We found that the *metabotropic glutamate receptor B* was highly abundant in *A. cincticrus* adult male, which is implicated in the regulation of aggression in honey bee^11^ and in the competition of reproduction in *Nasonia vitripennis* ^9^. The *progesterone* synthesized by the adrenocortical tissue was the highest in males, and may act indirectly on the brain after local conversion to other steroids that affect aggressive behavior. Allopregnanolone may be synthesized largely in males and is a neurosteroid that has been shown to play a role in regulating mammalian aggressive behavior^50,51^. These results of gene expression are consistent with the behavior that occurs in nature, i.e., *A. cincticrus* males exhibiting stronger aggressive behaviors against ants and other males to obtain food and win the mating chance. Similarly, *A. cincticrus* adults are generally larger in body size and inhabit in larger range of space suggesting they may exhibit stronger aggression than nymphs. Nymphs usually live underneath the stone, most often gregariously, likely consistent with their aggressive ability to avoid stress of fighting. Thus, it is not difficult to understand the camouflaging behavior in nymphs to protect themselves when facing danger or their enemies.

Insect predation is known to activate a series of mechanisms in order to locate prey, accompanied by sensitive identification of odor. Signal transmission from receptors to higher central nervous system rely on certain proteins, such as olfactory receptors (ORs) and OBPs^52^, that could discern stimulants of insects. For instance, green lacewing *Chrysopa pallens* use CpalOBP2 to detect and recognize the alarm pheromone in aphid species^5^. Hull *et al*.^53^ speculated that the plant bug *Lygus lineolaris* presented more *OBP*-like transcripts because of its broad host range. However, in pea aphid, there were relatively small number of *OBP*s owing to its parasitic lifestyle and specialized ecology^54^. Hekmat-Scafe *et al*.^55^ suggested that a moderate number of OBPs could act in a combinatorial manner with a moderate number of ORs to greatly increase the recognition power of an insect’s olfactory system. *ORs* were found highly expressed in male antennae of Asian corn borer due to its specific olfactory behavior and finding the emerging female^56^. Antennae-enriched CSPs may be involved in *C. pallens* identifying and binding volatile from pests, such as aphids, or pest-damaged plants^57^. Interestingly, the transcript abundance of these genes varied in different life stages of *A. cincticrus*. The transcripts were abundant in nymph and adult stages, suggesting a potential role in the detection and discrimination of odors. Surprisingly, greater sex bias was seen for some transcripts between genders, and *OBP*s were differentially expressed in nymph stages. The majority of these olfactory genes are highly expressed in the olfactory organ of female antennae than that of adult male, in contrast to their usual function for adult male to detect mates at long range, may be useful for females to detect pheromones released from ants in order to locate them accurately. Male-biased expression was observed in *ORs* in antennae, suggesting that they may mediate olfactory behavior specific to males, for example finding emerging adult female. The high expression of *OBPs* in *A. cincticrus* nymphs might suggest the unique functions in chemoreception. Compared with the egg stage, the nymphs and adults are active and had the need to detect odorants for their survival and reproduction in the environment.

## Methods

### Collection and colony maintenance

The colony of *A. cincticrus* used in this study was originally obtained from Yu County (39.9465°N, 114.9379°E), Hebei, China, then was maintained in a light incubator at 25 ± 2 °C, 60 ± 5% relative humidity (RH) under a 16 h: 8 h light/dark cycle. Specimens of eight life stages were collected as eggs, 1^st^ to 5^th^ instar nymphs, male and female adults. These samples were immediately frozen in liquid nitrogen and stored at −80 °C until RNA extraction.

### RNA isolation, cDNA library construction and Illumina sequencing

Total RNA was isolated using TRIzol reagent (Invitrogen, USA) according to the manufacturer’s protocols. RNA integrity was assessed using the RNA Nano 6000 Assay Kit in the Agilent Bioanalyzer 2100 system (Agilent Technologies, USA). The extracted RNA was used to establish a cDNA library for transcriptome sequencing using an Illumina Hiseq. 2500 sequencer platform at Biomarker Technologies Corporation (Beijing, China).

### Raw reads cleaning and quality control

In order to guarantee high-quality and clean reads, raw reads containing more than 5% unknown nucleotides and low-quality reads containing more than 50% of bases with a Q-value ≤ 20% were excluded from the sequence assembly. The clean reads were subjected to subsequent analyses and deposited in the NCBI SRA database under accession numbers: SRR5099968 (egg), SRR5099969-73 (1^st^ to 5^th^ instar nymphs), SRR5106081 (adult female) and SRR5106242 (adult male).

### Assembly and functional gene annotation

The clean reads were assembled using Trinity (http://trinityrnaseq.sourceforge.net/) with default assembly parameters. Clean reads with a certain overlap length were initially combined to form long fragments without N (named contigs). Related contigs were clustered using the TGICL software^58^ to yield unigenes (without N) that cannot be extended on either ends, and redundancies were removed to acquire non-redundant unigenes.

Subsequently, non-redundant unigenes were analyzed and annotated. Unigenes were mapped against the NCBI-NR, SwissProt and COG databases using the BLASTX algorithm with an e-value cut-off of 10^−5^ to retrieve functional annotations based on sequence similarity. High-priority databases (followed by NBCI-NR, SwissProt, and KEGG) were selected to determine the direction of the unigene sequences. According to the best alignment results, the coding sequences were generated. The ESTScan software^59^ was used to determine the sequence direction of the unigenes that could not be aligned to any of the above databases. Functional gene annotations were collected for all unigene sequences ≥ 150 bp using Blast2GO^60^. To determine the distribution of gene functions at the macro level, Blast2GO was used to retrieve the GO terms for each sequence by searching the NCBI-NR database. The WEGO software^61^ was used to plot the distribution of the GO functional classification of the unigenes. Functional pathway analysis was performed using the KAAS webserver^62^ from KEGG.

### Differential gene expression and trend analysis

Gene expression levels were calculated by RSEM^63^. The read counts were adjusted by the eDEGR program package through one scaling normalized factor. The DEGSeq package was used to analyze the gene expression difference between two groups. DEGSeq provided statistical routines for determining differential expression in unigenes using a model based on the negative binomial distribution. *P*-values were adjusted using the Benjamini-Hochberg’s approach for calculating the false discovery rate (FDR). Fragments per kilobase of exon per million fragments mapped (FPKM) was used to quantify the expression level of unigenes. Genes with an adjusted *p* < 0.05, FDR ≤ 0.01 and the absolute value of log_2_ Fold change (FC) ≥ 1 were set as the thresholds to identify the SDEGs. FC is the ratio of FPKM between the two samples. GO enrichment analysis of SDEGs was carried out based on an algorithm provided by GOstat^64^, with the complete annotation results set as the background. The *p*-value was approximated using the Chi-square test. Fisher’s exact test was used when any expected value was below 5. This program was implemented as a pipeline^65^.

STEM (Short Time-Series Expression Miner) software was used to profile gene expression time series and identify significant expression tendencies^66^ in these life stages based on FPKM values. Based on different signal density of genes under different situations, we identified a set of unique model expression tendencies. The raw expression values were converted into log_2_ ratios. Some unique profiles were further defined using a strategy for clustering short time-series gene expression data. Each model profile includes the actual or the expected number of genes. Fisher’s exact test and multiple-comparison tests were used to determine whether the significant profiles had higher probability than expected^67^.

### qRT-PCR validation

To validate the quality of the transcriptome data and estimate the relative expression, qRT-PCR was performed using the SYBR Premix Ex Taq (Takara, Japan) according to the manufacture’s protocols and a CFX96 real-time PCR detection system (Bio-Rad, USA). Primer sequences of selected genes were designed by DNAMAN and were listed in Table S10. Total RNA was extracted as described in the RNA isolation and cDNA was synthesized by Promega Goscript Reverse Transcription Systems (Fisher Scientific, USA). All qRT-PCR reactions had three biological and three technical replicates. The average threshold cycle (Ct) was calculated from values in all six replicates per sample. The *actin* gene was chosen as an endogenous control to normalize expression between different samples, as has been used in other insects^68^. The 2^−△△CT^ method^69^ was used to evaluate the relative gene expression. All data were statistically analyzed by independent sample students *t*-test using SPSS 17.0. The relative expression level of each unigene was presented as mean ± standard error (SE).

## Electronic supplementary material

Dataset 1

Supplementary information

## References

[1] Cao LM, Rédei D, Li H, Cai WZ (2014). Revision of the genus *Acanthaspis* Amyot & Serville (Hemiptera: Heteroptera: Reduviidae: Reduviinae) from China, with new records of species to adjacent countries. Zootaxa.

[2] Weirauch C (2006). Anatomy of disguise: camouflaging structures in nymphs of some Reduviidae (Heteroptera). Amer. Mus. Novi..

[3] Eisner T, Hicks K, Eisner M, Robson DS (1978). “Wolf-in-sheep’s-clothing” strategy of a predaceous insect larva. Science.

[4] Li H, Zhao GY, Cao LM, Xu K, Cai WZ (2010). Taxonomic and bionomic notes on the white spot assassin bug *Platymeris biguttatus* (Linnaeus) (Hemiptera: Reduviidae: Reduviinae). Zootaxa.

[5] Li ZQ (2015). Odorant-binding proteins display high affinities for behavioral attractants and repellents in the natural predator *Chrysopa pallens*. Comp. Biochem. Phys. A..

[6] Roeder T, Seifert M, Kähler C, Gewecke M (2003). Tyramine and octopamine: antagonistic modulators of behavior and metabolism. Arch. insect Biochem..

[7] Cohen AC (1993). Organization of digestion and preliminary characterization of salivary trypsin-like enzymes in a predaceous heteropteran *Zelus renardii*. J. Insect Physiol..

[8] Lorenz, K. Z. On Aggression. Harcourt, Brace & World, New York (1966).

[9] Tsai YJJ, Barrows EM, Weiss M (2014). Pure self-assessment of size during male-male contests in the parasitoid wasp *Nasonia vitripennis*. Ethology.

[10] Tsai YJJ, Barrows EM, Weiss M (2014). Why do larger and older males win contests in the parasitod wasp *Nasonia vitripennis*?. Anim. Behav..

[11] Alaux C (2009). Honey bee aggression supports a link between gene regulation and behavioral evolution. P. Natl. Acad. Sci. USA.

[12] Castro VD, Martínlópez M, Navarro JF (2010). Effects of Ly35740, a selective agonist for glutamate metabotropic receptors of group II, on aggressive behavior in mice. Univ. Psychol..

[13] Cohen AC (1995). Review: extra-oral digestion in predaceous terrestrial arthropod. Annu. Rev. Entomol..

[14] Castilla AM, Huey RB, Calvete JJ, Richer R, Al-Hemaidi AHM (2015). Arid environments: opportunities for studying co-evolutionary patterns of scorpion venoms in predator-prey systems. J. Arid Environ..

[15] Ambrose DP, Maran SPM (2000). Polymorphic diversity in salivary and haemolymph proteins and digestive physiology of assassin bug *Rhynocoris marginatus* (Fab.) (Het., Reduviidae). J. Appl. Entomol..

[16] George PJE, Ambrose DP (2001). Polymorphic adaptive insecticidal resistance in *Rhynocoris marginatus* (Fabr.) (Het., Reduviidae) a non-target biocontrol agent. J. Appl. Entomol..

[17] Edwards JS (1961). The action and composition of the saliva of an assassin bug *Platymeris rhadamanthus* Gaerst. (Hemiptera, Reduviidae). J. Exp. Biol..

[18] Evangelin G, Bertrand H, Muthupandi M, John WS (2014). Venomous saliva of non-haematophagous reduviid bugs (Heteroptera: Reduviidae): a review. Biolife.

[19] Yang CH, Yang PC, Li J, Yang F, Zhang AB (2016). Transcriptome characterization of *Dendrolimus punctatus* and expression profiles at different developmental stages. PLoS ONE.

[20] Li, L. T., Zhu, Y. B., Ma, J. F., Li, Z. Y. & Dong, Z. P. An analysis of the *Athetis lepigone* transcriptome from four developmental stages. *PLoS ONE* **8**, e73911 (2013). 10.1371/journal.pone.0073911 PMC3772797 24058501

[21] Ewen-Campen B (2011). The maternal and early embryonic transcriptome of the milkweed bug *Oncopeltus fasciatus*. BMC Genomics.

[22] Su SY (2009). Transcriptomic analysis of EGb 761-regulated neuroactive receptor pathway *in vivo*. J. Ethnopharmacol..

[23] Gibson JD, Niehuis O, Verrelli BC, Gadau J (2010). Contrasting patterns of selective constraints in nuclear- encoded genes of the oxidative phosphorylation pathway in holometabolous insects and their possible role in hybrid breakdown in *Nasonia*. Heredity.

[24] Erneux C, Govaerts C, Communi D, Pesesse X (1998). The diversity and possible functions of the inositol polyphosphate 5-phosphatases. Biochim. Biophys. Acta.

[25] Neurath H (1984). Evolution of proteolytic enzymes. Science.

[26] Horner V, Wolfner M (2008). Transitioning from egg to embryo: triggers and mechanisms of egg activation. Dev. Dynam..

[27] Li SW (2012). Transcriptome and gene expression analysis of the rice leaf folder, *Cnaphalocrosis medinalis*. PLoS ONE.

[28] Sparks ME, Shelby KS, Kuhar D, Gundersen-Rindal DE (2014). Transcriptome of the invasive brown marmorated stink bug, *Halyomorpha halys* (Stål) (Heteroptera: Pentatomidae). PLoS ONE.

[29] Ribeiro JMC, Pereira MEA (1984). Midgut glycosidases of *Rhodnius prolixus*. Insect Biochem..

[30] Feyereisen R (1999). Insect P450 enzymes. Annu. Rev. Entomol..

[31] Enayati AA, Ranson H, Hemingway J (2005). Insect glutathione transferases and insecticide resistance. Insect Mol. Biol..

[32] Staal GB (1975). Insect growth regulators with juvenile hormone activity. Entomology.

[33] Truman JW, Riddiford LM (1999). The origins of insect metamorphosis. Nature.

[34] Tabata T, Takei Y (2004). Morphogens, their identification and regulation. Development.

[35] de Celis JF, Garcia-Bellido A (1994). Roles of the notch gene in *Drosophila* wing morphogenesis. Mech. Dev..

[36] Hu JT, Chen B, Li ZH (2014). Thermal plasticity is related to the hardening response of heat shock protein expression in two *Bactrocera* fruit flies. J. Insect Physiol..

[37] Zhu J, Indrasith LS, Yamashita O (1986). Characterization of vitellin, egg-specific protein and 30 kDa protein from *Bomby*x eggs, and their fates during oogenesis and embryogenesis. Biochim. Biophys. Acta (BBA) Gen. Sujects.

[38] Sahayaraj K, Vinothkanna A (2011). Insecticidal activity of venomous saliva from *Rhynocoris fuscipes* (Reduviidae) against *Spodoptera litura* and *Helicoverpa armigera* by microinjection and oral administration. J. Venom. Anim. Toxins..

[39] Sahayaraj K, Kumara SS, Balasubramaniam R (2007). Prey influence on the salivary gland and gut enzymes qualitative profile of *Rhynocoris marginatus* (Fab.) and *Catamirus brevipennis* (Serville) (Heteropetera: Reduviidae). J. Entomol..

[40] Law JH, Dunn PE, Kramer KJ (1977). Insect proteases and peptidases. Adv. Enzymol..

[41] Arnott G, Elwood RW (2009). Assessment of fighting ability in animal contests. Anim. Behav..

[42] Briffa M (2008). Decisions during fights in the house cricket, *Acheta domesticus*: mutual or self assessment of energy, weapons and size?. Anim. Behav..

[43] Brown WD, Smith AT, Moskalik B, Gabriel J (2006). Aggressive contests in house crickets: size, motivation and the information content of aggressive songs. Anim. Behav..

[44] Stevenson PA, Schildberger K (2013). Mechanisms of experience dependent control of aggression in crickets. Curr. Opin. Neurobiol..

[45] Hartley CS, Matthews RT (2003). The effect of body size on male-male combat in the parasitoid wasp *Melittobia digitata* Dahms (Hymenoptera: Eulophidae). J. Hymenopt. Res..

[46] Barry KL, Kokko H (2010). Male mated choice: why sequential choice can make its evolution difficult. Anim. Behav..

[47] Bubak AN, Grace JL, Watt MJ, Renner KJ (2014). & Swallow, J. G. Neurochemistry as a bridge between morphology and behavior: perspectives on aggression in insects. Curr. Zool..

[48] Wang L, Dankert H, Perona P, Anderson DJ (2008). A common genetic target for environmental and heritable influences on aggressiveness in Drosophila. P. Natl. Acad. Sci. USA.

[49] Grohmann L (2003). Molecular and functional characterization of an octopamine receptor from honeybee (*Apis mellifera*) brain. J. Neurochem..

[50] Stevenson PA, Hofmann HA, Schoch K, Schildberger K (2000). The fight and flight responses of crickets depleted of biogenic amines. J. Neurobiol..

[51] Fish EW, DeBold JF, Miczek KA (2002). Aggressive behavior as a reinforcer in mice: activation by allopregnanolone. Psychopharmacology.

[52] Fan J, Francis F, Liu Y, Chen JL, Cheng DF (2011). An overview of odorant-binding protein functions in insect peripheral olfactory reception. Genet. Mol. Res..

[53] Hull JJ, Perera OP, Snodgrass GL (2014). Cloning and expression profiling of odorant-binding proteins in the tarnished plant bug, *Lygus lineolaris*. Insect Mol. Biol..

[54] Zhou JJ (2010). Genome annotation and comparative analyses of the odorant-binding proteins and chemosensory proteins in the pea aphid *Acyrthosiphon pisum*. Insect Mol. Biol..

[55] Hekmat-Scafe DS, Scafe CR, McKinney AJ, Tanouye MA (2002). Genome-wide analysis of the odorant-binding protein gene family in *Drosophila melanogaster*. Genome Res..

[56] Zhang TT (2015). Male- and female- biased gene expression of olfactory-related genes in the antennae of Asian corn borer, *Ostrinia furnacalis* (Guenée) (Lepidoptera: Crambidae). PLoS ONE.

[57] Li ZQ (2013). First transcriptome and digital gene expression analysis in Neuroptera with an emphasis on chemoreception genes in *Chrysopa pallens* (Rambur). PLoS ONE.

[58] Pertea G (2003). TIGR Gene Indices clustering tools (TGICL): a software system for fast clustering of large EST datasets. Bioinformatics.

[59] Iseli C, Jongeneel CV, Bucher P (1999). ESTScan: a program for detecting, evaluating, and reconstructing potential coding regions in EST sequences. Proc. Int. Conf. Intell. Syst. Mol. Biol..

[60] Conesa A (2005). Blast2GO: a universal tool for annotation, visualization and analysis in functional genomics research. Bioinformatics.

[61] Ye J (2006). WEGO: a web tool for plotting GO annotations. Nucleic Acids Res..

[62] Moriya Y, Itoh M, Okuda S, Yoshizawa AC, Kanehisa M (2007). KAAS: an automatic genome annotation and pathway reconstruction server. Nucleic Acids Res..

[63] Li B, Dewey CN (2011). RSEM: accurate transcript quantification from RNA-Seq data with or without a reference genome. BMC Bioinformatics.

[64] Beißbarth T, Speed TP (2004). GOstat: find statistically overrepresented gene ontologies within a group of genes. Bioinformatics.

[65] Chen S (2010). *De novo* analysis of transcriptome dynamics in the migratory locust during the development of phase traits. PLoS ONE.

[66] Ernst J, Bar-Joseph Z (2006). STEM: a tool for the analysis of short time series gene expression data. BMC Bioinformatics.

[67] Ramoni MF, Sebastiani P, KohaneI S (2002). Cluster analysis of gene expression dynamics. P. Natl. Acad. Sci. USA.

[68] Zou DY (2013). Nutrigenomics in *Arma chinensis*: transcriptome analysis of *Arma chinensis* fed on artificial diet and Chinese oak silk moth *Antheraea pernyi* pupae. PLoS ONE.

[69] Livak KJ, Schmitten TD (2001). Analysis of relative gene expression data using real-time quantitative PCR and the 2^−△△CT^ method. Methods.

