# Supplementary material for: Temporal transcriptomic profiling of the ant-feeding assassin bug Acanthaspis cincticrus reveals a biased expression of genes associated with predation in nymphs
Source: Sci Rep. 2017 Oct 4;7:12691. doi: 10.1038/s41598-017-12978-0 (PMC5627237; doi:10.1038/s41598-017-12978-0)
Supplement: Supplementary file 2 — Supplementary information [file 41598_2017_12978_MOESM2_ESM.pdf]

Temporal transcriptomic profiling of the ant-feeding assassin bug *Acanthaspis cincticrus* reveals a biased expression of genes associated with predation in nymphs

Fei Kou[1], Hu Li[1], Shujuan Li[2], Huaizhu Xun[1], Yinqiao Zhang[1], Ziqiang Sun[1], Xuguo Zhou[3] and Wanzhi Cai[1§]

[1] Key Laboratory of Pest Monitoring and Green Management, Ministry of Agriculture Department of Entomology, China Agricultural University, Beijing 100193, China

[2] Maricopa Agricultural Center, University of Arizona, Maricopa, AZ 85138, USA

[3] Department of Entomology, University of Kentucky, Lexington, KY 40546-0091, USA

[§] Correspondence and requests for materials should be addressed to W. C. (caiwz@cau.edu.cn)

Supplementary Information: Figures S1-S6 and Tables S1-S10.

Supplementary Figure S1. Functional categories of the unigenes in GO database. Unigenes were annotated in three categories: cellular component, molecular function and biological process.

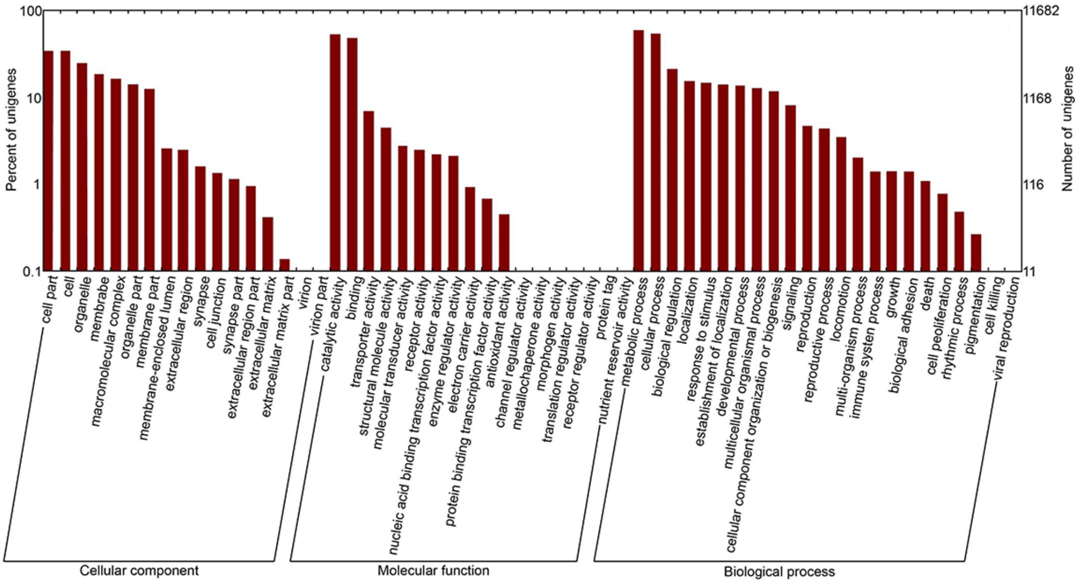

Supplementary Figure S2. COG categories of the unigenes. The name of each classification (A to Z) is provided on the right side of the figure.

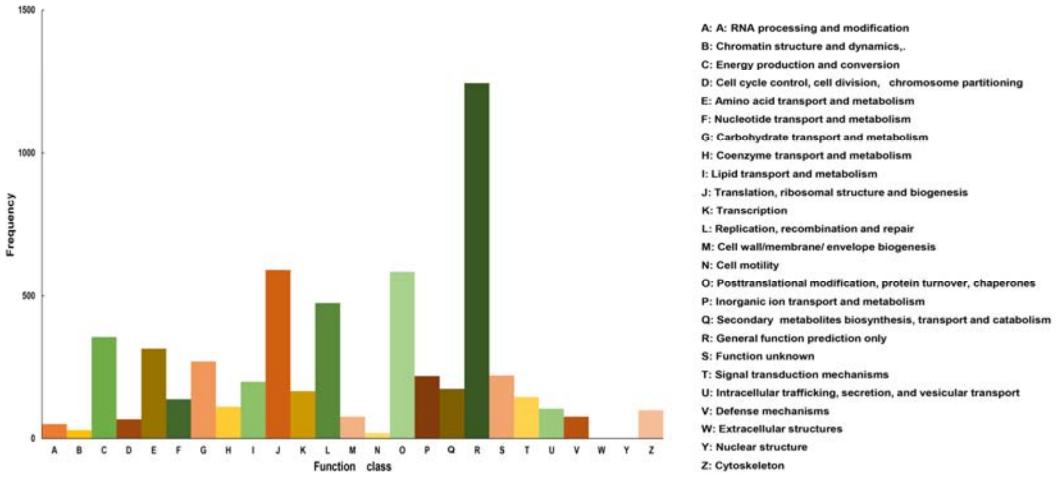

Supplementary Figure S3. Heatmap of gene expression profiles using SDEGs in Table S4. The color scales represent the $\log_2$-transformed FPKM values of eight life stages.

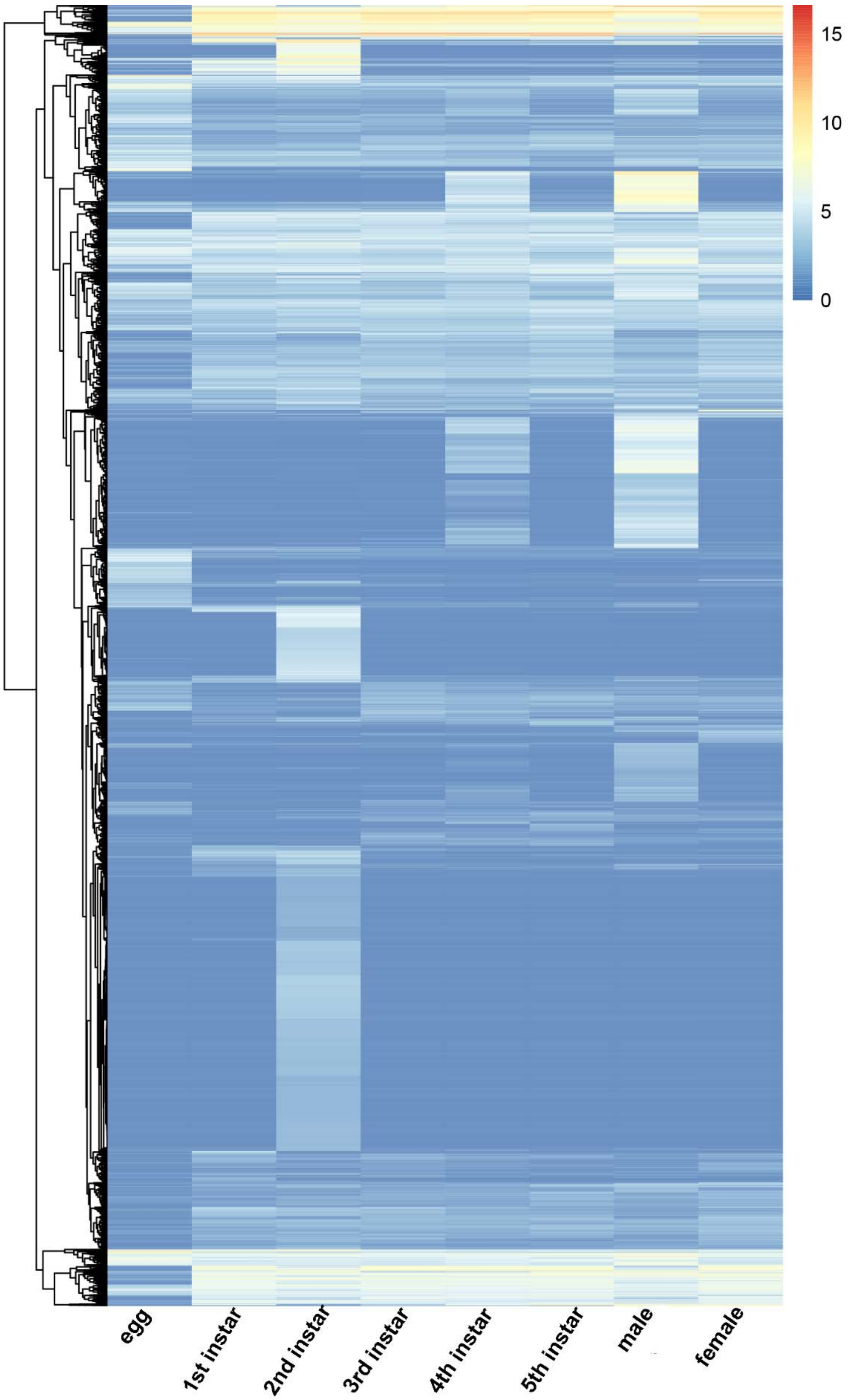

Supplementary Figure S4. Veen diagram showing the distribution of SDEGs between the five nymph stages.

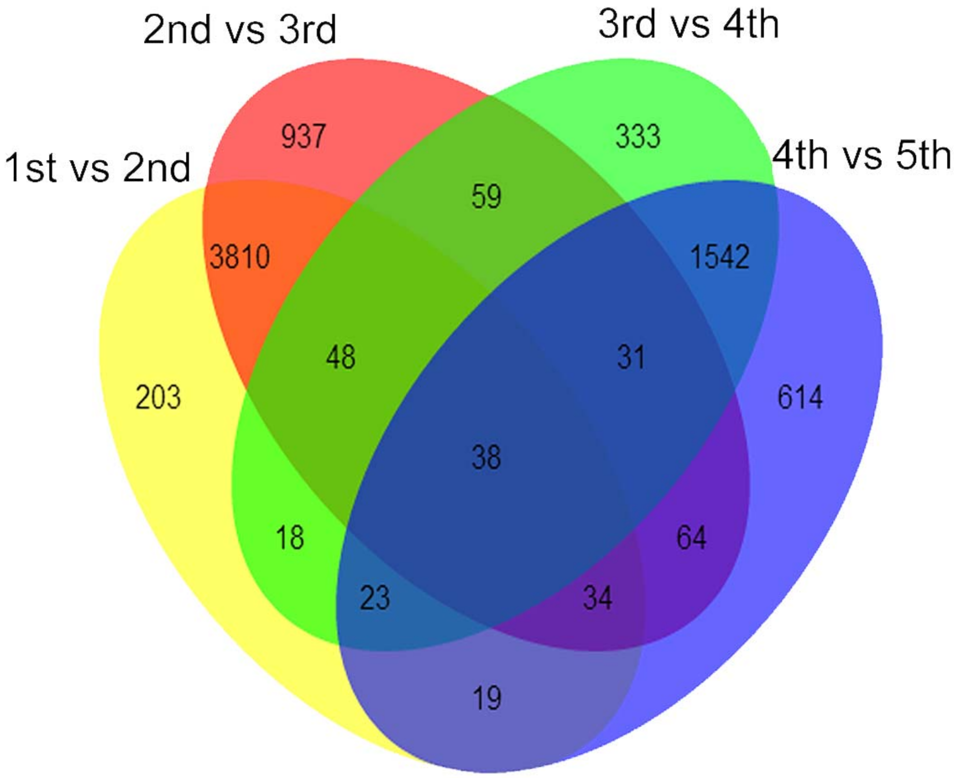

Supplementary Figure S5. The representative GO terms enriched by SDEGs between life stages. (A) Biological process. (B) Cellular component. (C) Molecular function. Numbers of the upregulated and downregulated SDEGs were shown.

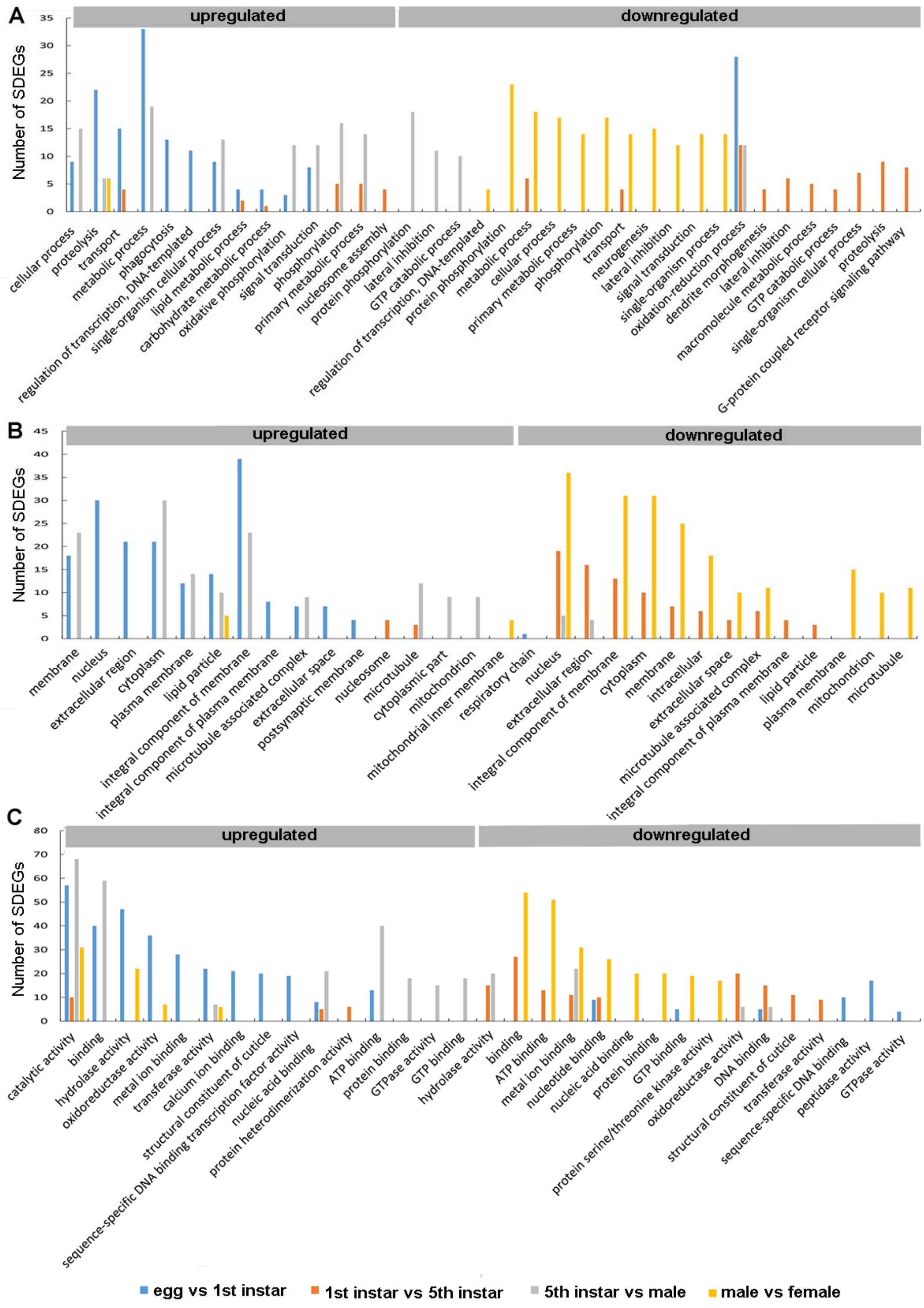

Supplementary Figure S6. Expression patterns of 13,479 SDEGs for the eight life stages based on model profiles. Each square was a model expression profile and colored squares represented significant enrichment ($p < 0.05$). The upper number in the profile square was the model profile number and the lower number was the *p*-value correspond to the differential gene expression test. "1-8" respectively represented the egg, 1st to 5th instar nymphs, male and female adults.

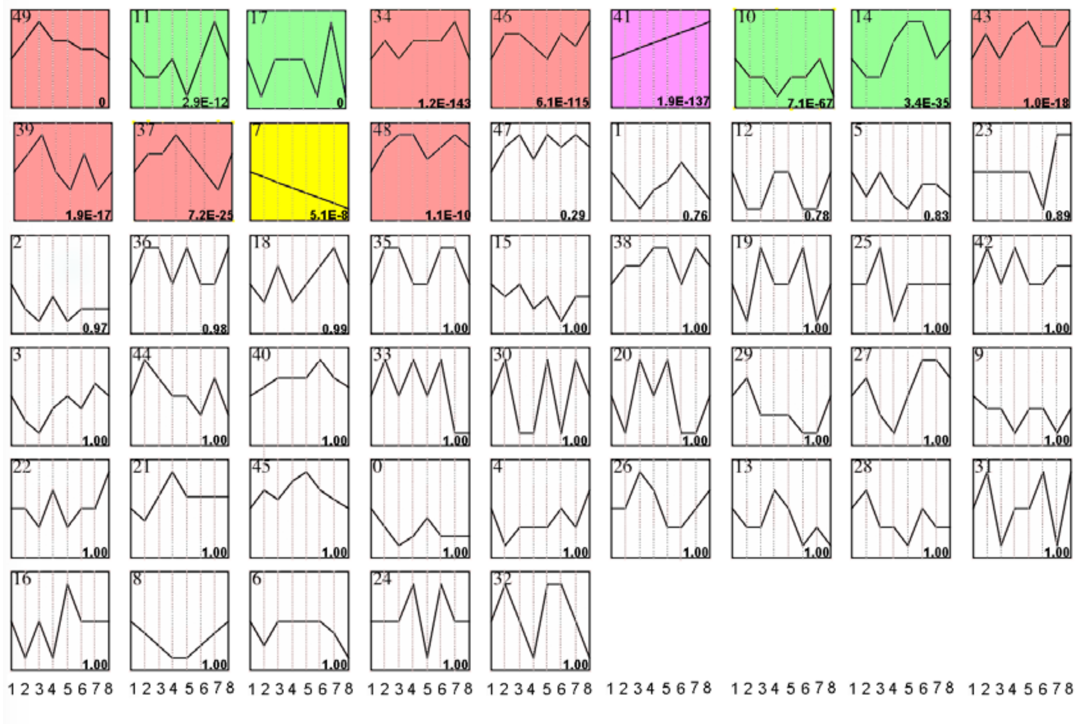

Supplementary Table S1. Overview of the sequencing results.
Please see the supplementary Excel file (Supplementary Dataset 1).

Supplementary Table S2. The KEGG pathway analysis of *A. cincticrus* transcriptome.
Please see the supplementary Excel file (Supplementary Dataset 1).

Supplementary Table S3. The SDEGs between nine comparisons.
Please see the supplementary Excel file (Supplementary Dataset 1).

Supplementary Table S4. The FPKM of SDEGs between different life stages.
Please see the supplementary Excel file (Supplementary Dataset 1).

Supplementary Table S5. Genes identified associated with the embryonic development, heat shock protein, vitellogenin, sperm-related, cuticular protein, juvenile hormone binding protein, ecdysone-induced protein, cytochrome P450s, glycosidases, transport oxidoreductase, histone 2A, notch, and wnt.
Please see the supplementary Excel file (Supplementary Dataset 1).

Supplementary Table S6. The number of SDEGs in different comparisons.
Please see the supplementary Excel file (Supplementary Dataset 1).

Supplementary Table S7. GO enrichment analysis in each comparison.
Please see the supplementary Excel file (Supplementary Dataset 1).

Supplementary Table S8. KEGG enrichment analysis in each comparison.
Please see the supplementary Excel file (Supplementary Dataset 1).

Supplementary Table S9. The SDEGs putatively related to predation identified in transcriptome.
Please see the supplementary Excel file (Supplementary Dataset 1).

Supplementary Table S10. Primers used in qRT-PCR for the validation of SDEGs.
Please see the supplementary Excel file (Supplementary Dataset 1).
